# Microlearning for patient safety: Crew resource management training in 15-minutes

**DOI:** 10.1371/journal.pone.0213178

**Published:** 2019-03-07

**Authors:** Benedict Gross, Leonie Rusin, Jan Kiesewetter, Jan M. Zottmann, Martin R. Fischer, Stephan Prückner, Alexandra Zech

**Affiliations:** 1 Institute for Emergency Medicine and Management in Medicine, University Hospital LMU Munich, Munich, Germany; 2 Institute for Medical Education, University Hospital LMU Munich, Munich, Germany; University of South Australia, AUSTRALIA

## Abstract

**Objectives:**

We sought to establish the feasibility of chunking crew resource management (CRM) training into micro-size interventions and to compare different training approaches in the context of micro-learning.

**Design:**

We evaluated whether participants in micro-learning CRM activities achieved learning objectives following training. In a between-subjects design, groups were observed for behaviour during a simulation that was part of a 15-minute modular intervention and tested for recollection afterwards.

**Participants:**

The 129 participants recruited for this study were medical students, who already had relevant experience treating patients.

**Interventions:**

The experimental setting involved three 5-minute components: video, simulation, and debriefing. Different groups viewed videos involving different didactic concepts: one group observed a videotaped concrete example of a medical care team applying a CRM tool (example group), and one group observed a videotaped lecture on the same topic (lecture group).

**Main outcome measures:**

All simulations were videotaped and coded in detail for the occurrence of and time spent engaging in team behaviour and medical care. Questionnaires were administered before, immediately after, and two weeks after the intervention. We compared the groups’ behaviour during the simulation (team cooperation and medical care), retention of knowledge from the training content, and results of the evaluation.

**Results:**

Both groups exhibited most of the behaviours included in the content of the instructional videos during the simulations and recollected information 2 weeks later. The example group exhibited significantly more of the training content during the simulation and demonstrated better retention 2 weeks later. Although the example group spent more time on team coordination, there was no difference in the number of executed medical measures.

**Conclusion:**

Delivering CRM training in chunks of relatively short and highly standardised interventions appears feasible. In this study, the form of didactical presentation caused a difference in learning success between groups: a traditional lecture was outperformed by an instructional video demonstrating a practical example.

## Introduction

Team training is considered a crucial factor for patient safety in healthcare [1]. Crew resource management (CRM) has become a leading example of such training. CRM holds its roots in aviation where it was developed over the last four decades to address failures of interpersonal communication, decision-making, and leadership. Modern CRM involves an array of tools for error management in high-risk environments [2].

### Structured briefing models

Representative tools in CRM include structured briefing models, such as SBAR (Situation; Background; Assessment; Recommendation) [3–5] and FOR-DEC (Facts; Options; Risks and Benefits; Decision; Execution; Check) [6]. Such acronyms support communication and decision making, particularly during critical situations. However, they may be perceived as time-consuming in stressful situations and users may tire of using these formal communication processes where an unambiguous procedure exists or a single solution is clearly evident [6]. These potential barriers to acceptance should be considered for healthcare teams, where–unlike aviation crews–staff configurations are more heterogeneous, with a number of subspecialties involved and frequently changing while critical situations may arise multiple times during a typical shift [7]. Tools that are perceived as unwieldy will not gain acceptance under such conditions. Weller et al. developed a structured call-out tool based on SBAR that is individualised for the daily routine of medical teams: Stop; Notify; Assessment; Plan; Priorities; Invite ideas (SNAPPI) [8]. This acronym was designed to improve information-sharing within medical teams during crisis situations and to create a shared understanding of the clinical situation. This call-out tool might be relevant within a wider variety of situations because it is less formal than pre-existing acronyms that stem from an aviation or military context. Weller et al. were able to teach anaesthetists using SNAPPI through a 15 minute video-based educational intervention.

### The science of training

More than a decade ago, Salas and colleagues raised several critical concerns regarding the qualification of CRM training faculty, the design of training, the didactical concepts underlying the system, and the embedding of the tools in an organisational context. They questioned the “science of training”, proposing that the way training is designed, delivered, and implemented can greatly influence its effectiveness [9–11]. This critique remains relevant today, highlighting potential shortcomings of CRM in healthcare. However, multiple recent reviews have concluded that the content and characteristics of team training [12,13], including CRM training [14,15], were seldom reported in a reproducible way. These findings indicate that more is known about the “if” than the “how”, in terms of the way training works.

Rather than theoretical knowledge transfer, CRM training typically focuses on equipping professionals with practical skills for working in teams, communicating effectively, and making decisions in real-world critical situations. Learners usually first develop procedural knowledge to understand information about complex system components, states, and their functions. According to the human factors research that underlies aviation CRM, such “schemata” contain sequences of appropriate actions for different types of tasks [16]. For tasks including social interactions, educational psychologists speak of “collaboration scripts” serving as a guide for the distribution of roles and activities among team members [17]. Learners can develop such collaboration scripts and schemata through training and experiences in a simulated environment involving authentic situations that are characteristic of their later professional practice [16,18].

**Fig 1:**
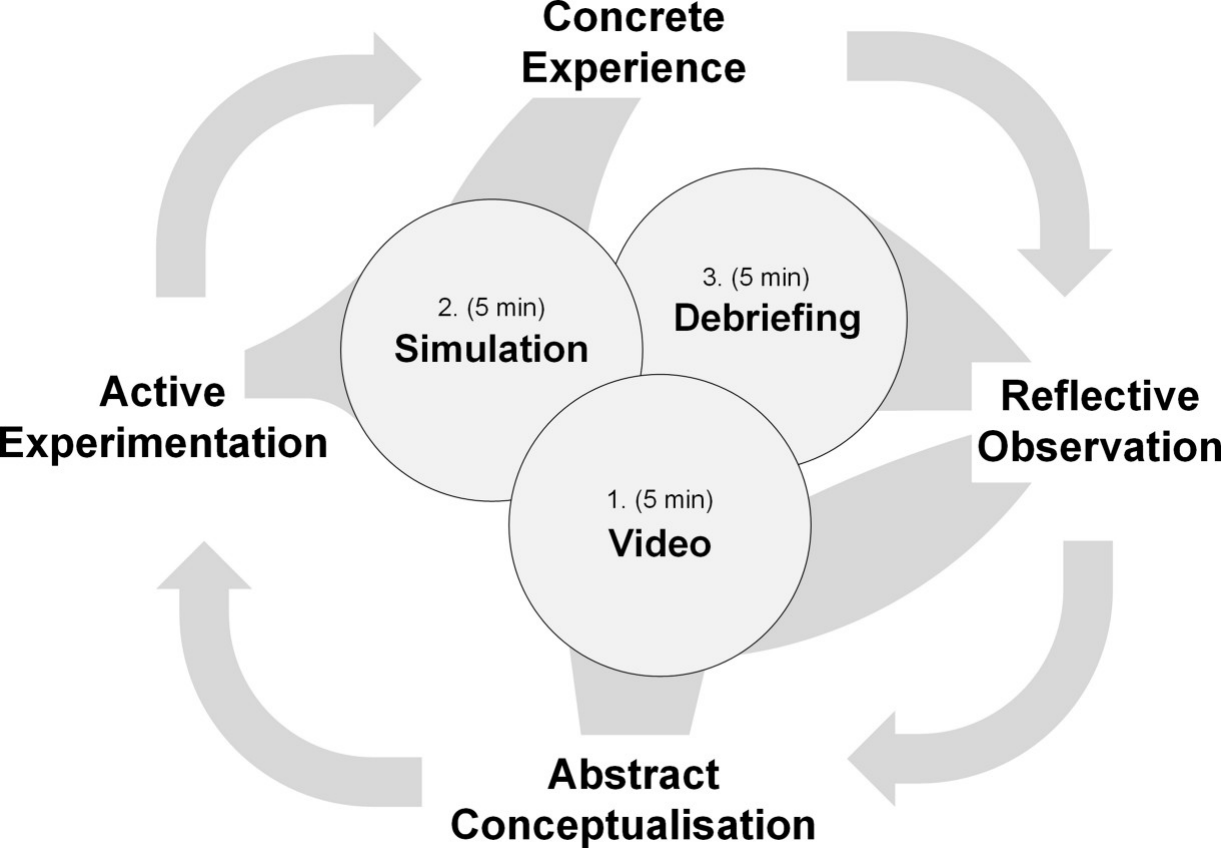
The intervention was designed to run through one ELT cycle in a 15-minute period. The figure demonstrates how the three parts of the intervention (1. Video, 2. Simulation, 3. Debriefing) touched all four bases of the ELT cycle.

Experiential Learning Theory (ELT) provides a theoretical framework to design experience-based training interventions. This framework conceptualises learning as a recursive process of experiencing, reflecting, thinking, and acting [19]. ELT describes a “cycle of experiential learning” consisting of four modes. The first two modes, Concrete Experience and Abstract Conceptualisation, allow learners to grasp experience, whereas the latter modes, Reflective Observation and Active Experimentation, support learners to convert experience into knowledge. Fig 1 shows a visualisation of the ELT cycle. Accordingly, CRM training can be designed as a spiral of experiences, observations, and reflections which nourish a learner’s pool of scripts and schemata for critical situations.

### Challenges to traditional training formats

Applying CRM training in practice involves overcoming the barriers involved in traditional educational formats, which can arise from economic and demographic factors. In a systematic review of the literature regarding CRM training in healthcare, training formats were typically found to be interventions delivered in a traditional attendance-based seminar format extending over 1 day with approximately five to 15 participants [20]. Such training durations of several hours can place a burden on health care institutions that already have to cope with limited staffing levels [21]. Scheduling a whole team for training requires not only a direct investment but also additional opportunity costs related to the necessity to keep facilities operational with a second team [22]. This can result in a conflict of interest between allocating staff to training for patient safety and upholding an appropriate level of service.

However, demographic trends additionally increase the need to adapt academic and vocational education to the changing ways in which medical students and junior staff acquire new skills. A growing cohort of “digital learners” [23] or “new millennium learners” [24] have new habits and expectations regarding education. These learners are reported to prefer receiving information quickly, acquiring knowledge independently from senior staff, utilising multiple sources and digital content, and spending less time in books and more time dealing with medical cases [24,25]. Moreover, students interacting with learning content is now the norm, not the exception. The impact of such new developments in education, in terms of both technology and audience, presents a particular challenge for traditional higher education systems, where lectures may no longer be the only or best way of transferring knowledge [26,27].

### Getting the most out of a short intervention

One potentially helpful response to new learning requirements and economic restraints is to chunk the learning process into smaller episodes, skill elements, or “knowledge nuggets”, which involve minimal time consumption and operating expense, and can be part of a modular curricular setting. Such educational concepts have been labelled “microlearning” [28], although no conclusive definition or uniform body of research is currently available. We have adopted the term here, to describe a very concise intervention of teaching a small group of students within a relatively short period of 5 to 15 minutes and then giving and taking feedback on the performances [29]. In management and executive education, learning in “rapid bite-sized chunks” has become a successful product in the corporate training market [30]. In addition, for schools and universities, implications of modularisation in small units of knowledge and chunking of contents are currently receiving attention [27,31].

One example of a standardised and modularised CRM curriculum is the TeamSTEPPS 2.0 program of the US-American Agency for Healthcare Research and Quality [32]. The TeamSTEPPS teaching material includes short videos in which actors demonstrate the application of good teamwork in real-world situations. The advantages of such formats have been advocated by Clay-Williams et al. who designed CRM modules of 2-hour workshops as alternatives to full-day seminars that can be problematic and impractical for the “time-poor clinician”.[33]

### Rationale for the current study

The current study was conducted to extend the approach of teaching a CRM tool during a self-contained intervention taking less than 15 minutes, as it has already been established by Weller et al.’s SNAPPI tool and the modules of the TeamSTEPPS program. Thus, the research project sought to further adopt the concept of microlearning to create a modular and lightweight CRM training program substantiated on the Experiential Learning Theory. The intervention concept is designed to be delivered in a standardised module that is largely independent of trainer personality and qualifications, and short enough to be implemented in the daily routine of healthcare organisations.

Specifically, this study focused on “how” to train. When intervention time is restricted to only a short interval to acquire CRM skills, we wanted to examine the importance of the instructional approach for theory input. For this purpose, the performance of an instructional video showing a practical example was compared with a traditional academic lecture.

### Research question

To generally demonstrate the feasibility of microlearning for CRM training, we examined if it would be possible to develop content for a 15-minute intervention that would enable learners to achieve high scores with respect to demonstrating learning objectives.

The specific research question for our experiment was: To what extent does the instructional approach during a module (instructional video vs. lecture) affect immediate learning outcomes and retention of knowledge?

To control for possible negative effects of the CRM intervention, we examined if an increased emphasis on social team processes would concur with technical measures of medical care.

## Methods

Comparing example-based video content to lecture-based video content involved a between-subjects design. Two experimental groups completed a 15-minute intervention composed of either an instructional video or videotaped lecture, followed by simulation and debriefing. The key difference between groups was the didactical concept of the videos: one group viewed a concrete example of a medical care team applying a CRM tool (example group) whereas another group viewed a videotaped lecture on the same topic (lecture group). See evaluation design section for an overview of the three parts of intervention and the evaluation methods.

### Ethics

The human subjects research activities were reviewed and approved by the ethics committee of the medical faculty at LMU Munich (review number 227–16), written consent was obtained from all participants. The individuals depicted in the transcripts of the videos (S1 File) are professional actors (example video) or member of faculty (lecture video) and gave written informed consent (as outlined in PLOS consent form) to publish screenshots of the videos.

### CRM Tool

In this study, we developed a CRM tool to deliver distinctive educational content. The tool needed to be unique in concept and wording, meaning that participants could not be familiar with it and biased by prior theoretical input or practical experience. The authors, consisting of a group of experts from medicine (LR), psychology (JK, AZ), educational (JZ) and management sciences (BG), developed a cohesive three-step protocol for team briefings. It was inspired by structured briefing models, such as SBAR and FOR-DEC, as well as Weller et al.’s SNAPPI method [8]. We intended to create an even more lightweight tool compared to the established, but rather formal, acronyms to accommodate the fast-paced and often recurring critical situations healthcare teams face on a daily basis.

We named the proposed CRM tool “Team Check” to provide a distinctive key phrase that would be observable during a simulation. The purpose of the protocol is to first gather and align distributed information in a team, then mutually agree on a plan for the next steps, and finally, to distribute roles and activities among team members. See Table 1 for a description of the Team Check procedure.

**Table 1:** Team check protocol.

Team Check	
When to use? In situations when all resources of a team must be focused. How to use? The code word “Team Check” marks the begin of the briefing. The protocol is summarised below. Important: After every step, ask the team if everybody is OK!	
Code word	“Team Check”
(What?)	Describe the problem • What is the situation? “Correct?”
(How?)	Plan and prioritise • How do we solve the problem? • What are next steps? “Correct?”
(Who?)	Delegate • Who will take over which task or role? “Do we consent?”
Code word	“Go”

A similar communication tool is propagated as “10-for-10 principle” in the medical simulation community [34]. It describes a form of call-out team briefing in order to assess the situation thoroughly and select the correct actions as a process of expert decision-making. See Table 2 for a description of the 10-for-10 principle. In comparison, the Team Check protocol is designed to be more focussed, but it still covers all the same aspects. Both tools aim at improving communication for better teamwork and crisis management [35]. The 10-for-10 principle was therefore selected as intervention for the comparison group in this study.

**Table 2:** The 10-for-10 principle.

10-seconds-for-10-minutes principle according to Rall et al. [34]	
When you see a patient in a critical condition, take your time, do not make a diagnosis and start treatment within a fraction of a second, but take a deep breath and then a formal team time-out.	
Problem?	Ask yourself and all your team members, ‘What is the biggest problem right now?’–‘What is the most dangerous aspect of the problem?’
Opinions?	Clarify the above with all available team members.
Facts?	Gather available information.
Plan?	Using input from the team, make a treatment plan. This includes the plan as well as the sequence of actions.
Distribute?	Distribute the workload by assigning tasks and responsibilities.
Check!	Before diving into work, involve all team members again to encourage them to raise any further concerns or suggestions for improvement or refinement.

### Intervention

The experimental setting contained three 5-minute components: video, simulation, and debriefing. The intervention was designed to give participants the opportunity to touch all bases of the ELT cycle: watching the video initiates Abstract Conceptualisation and Reflective Observation. Participants were then prompted to learn through Active Experimentation and Concrete Experience during the simulation. Finally, participants experienced Reflective Observation again during the debriefing session (see Fig 1). The whole setting and procedures were pre-tested in nine complete runs to train the faculty and optimise the simulation scenario and timings. The three components of the intervention are described below.

#### 1. Video

Participants in the example group watched an instructional video showing a healthcare team applying the Team Check procedure. The video was created in the professional style of a documentary television series in the first half, then changed to a more educational style by recapitulating each step of the Team Check protocol to elaborate on its purpose and application. The video ended with short interviews with the actors describing the benefits of the Team Check protocol and why it is useful in their particular role. The actors illustrated different role models: An experienced female physician as the team leader, a senior male nurse, and a female medical student.

The lecture group watched a video of the same length showing a recorded lecture about the danger of uncoordinated teamwork during critical situations in healthcare. To avoid these deficits, the “10-for-10 principle” was presented to this group as team briefing tool. The codeword “Stop” was introduced to initiate a step-back method for all team members to reassess the situation and bundle information. See supplementary material (S1 File) for a transcript of the videos. To avoid bias in the lecture group, the lecturer was selected from the faculty of our university hospital, who regularly gives human factors trainings and is well-rated in evaluations. The lecturer was allowed 5 weeks to prepare before the videotaping, and gave a typical presentation that is part of the CRM education curriculum for medical students at the university hospital.

Although the videos for both groups contained similar theoretical content, the video viewed by the example group involved dramaturgy designed to address the ELT areas of Abstract Conceptualisation and Reflective Observation, whereas the lecture group received a traditional lecture-based form of abstract transfer of knowledge.

#### 2. Simulation

After watching the video, participants of both groups underwent an identical simulated scenario of a standard situation in an emergency ward. In this simulation, a patient was admitted by ambulance after syncope of unclear origin. Participants were assigned to different roles, as described below. All participants were instructed as medical students assisting in a particular department as part of their education. One was from the surgical program, one was from the internal department of the hospital, and the third participant was briefed to assist in the ambulance service. All participants were given typical working clothes and provided with a folder of documents. The simulation was arranged so that each participant found documentation in their folder, indicating that their department had previously seen the patient. The ambulance role had the paramedic protocol for the day, and the internal and surgical roles each had medical records from their department at which the patient was previously seen, but on different days and with different diagnostic results.

Before the simulation began, all participants were briefly familiarised with the setting, equipment, and simulation mannequin (Sim Man 3G, Leardal, Stavanger, Norway). Participants had access to an array of medical devices that are typical for an examination room, including a stethoscope, blood pressure cuff, diagnostic penlight, pulse oximeter, NaCl infusion, and oxygen masks. An electrocardiogram (ECG) monitor was always attached to the patient and running.

**Fig 2:**
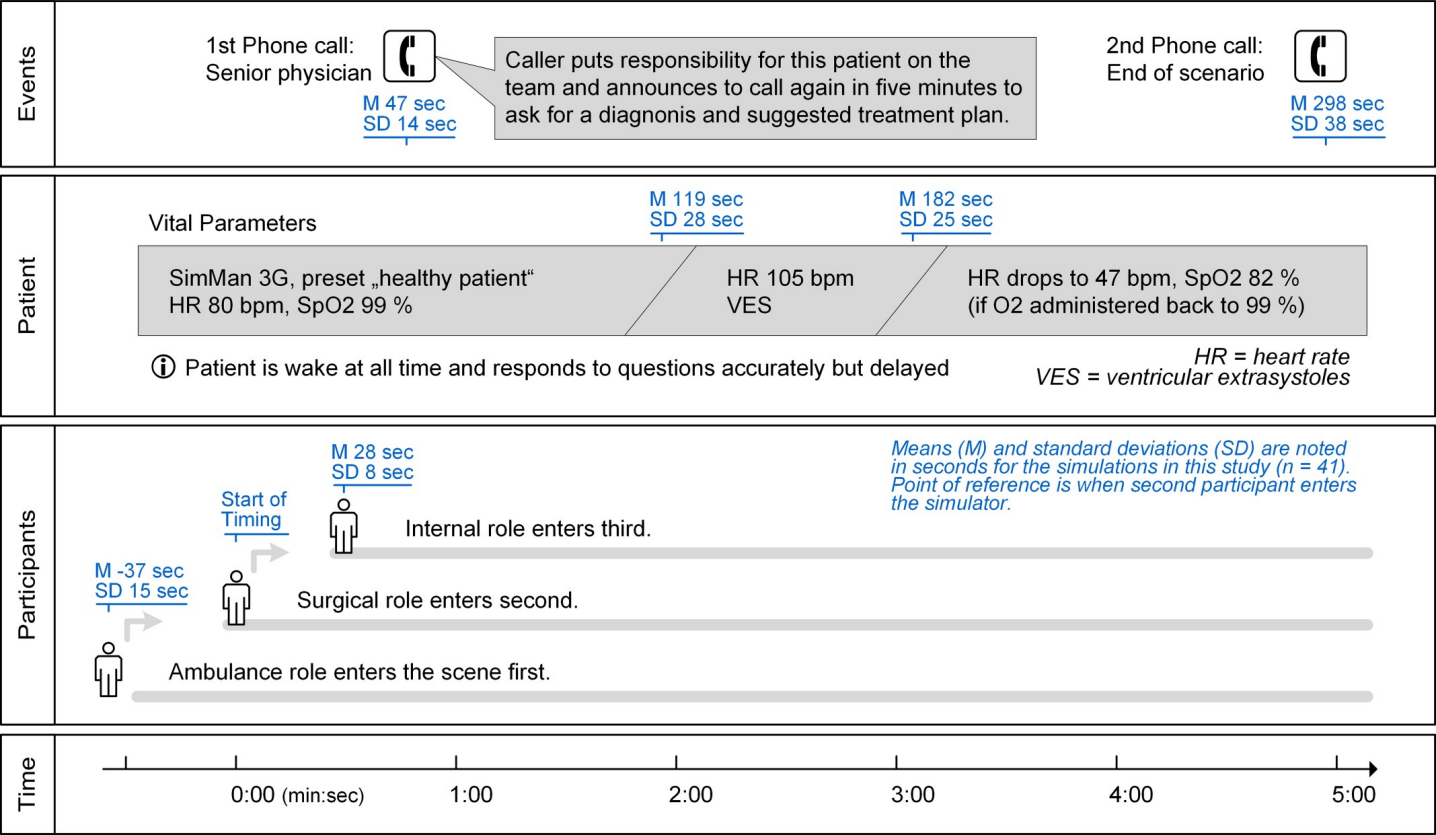
Sequence and timings for the simulated scenario. The Flowchart demonstrates the scenarios’ standardised course of events. Patient conditions and external events followed a predefined schedule, participants entered the scenario with staggered timings. The effective timings as surveyed during the simulations (n = 41) are reported as mean (M) and standard deviation (SD).

The simulation followed a predefined sequence, as described in Fig 2. To create a situation of distributed information involving the need for team processes, participants were brought into the scenario with staggered timing. The medical conditions were arranged so that a critical emergency never occurred (e.g., resuscitation). The patient was awake at all times and responded to the participants. Nonetheless, the situation demanded a senior physician’s attention, and amongst all the available measures of medical care, the application of supplemental oxygen and a call for assistance (“call for help”) represent the essential and urgent actions in the situation.

To avoid a bias, the scenario had substantial differences to the example situation presented in the Team Check video. It was designed to have no similarities either in team roles or in clinical details: during the simulation the patient was awake and able to respond at all times, showing a critical change in vital parameters, being admitted in this moment (with ambulance staff still present), anamnesis and documented information available but shared amongst departments, and a hierarchy-free team of juniors form internal and chirurgical department and ambulance service. The Team Check video in contrast featured a team constellation of a resident, a senior nurse and a student, while the patient was admitted several hours ago, with unknown anamnesis, being somnolent and not responsive, with advanced diagnostic processes already started.

#### 3. Debriefing

After the simulation, participants left the simulator and were invited to a debriefing in the next room. Following debriefing guidelines, participants were asked about their experience during the simulation and what strategies helped them cope with challenges, using open questions. If no keywords from the video were mentioned (like “Team Check” for the example group and respectively “10 for 10” or “Stop” for the lecture group), it was explicitly asked if the video was helpful. All participants were complimented for their performance in the simulation and were given the chance to ask questions, so that all participants were released with positive feedback.

### Recruiting strategy

Participants were recruited via newsletter and social media from medical students at an advanced stage of their degree at two local universities. Inclusion criteria were that the students had already completed their preliminary medical examination or gathered relevant experience treating patients during an earlier occupation (e.g., working as nurse or paramedic). This group of participants was able to be immersed in the clinical scenario but unlikely to have already been exposed to CRM or leadership training, rather than residents or practicing physicians. Participants were randomly invited in groups of three which were alternately assigned to A or B intervention.

### Evaluation design

All simulations were videotaped and coded afterwards in terms of team cooperation and medical care. Details of video recordings processing are reported in the data analysis section below. Additional data were collected with questionnaires at three points in time: before participants saw the video (t0), after the debriefing (t1), and 2 weeks later (t2). The t0 questionnaire contained demographic items and asked for prior experiences. In the t1 questionnaire, participants evaluated the training on a 6-point Likert scale (items depicted in S2 File) and were asked to note the video elements they remembered in a free text field. With an offset of two weeks after the intervention, participants were sent a link to an online questionnaire via e-mail to observe the sustainability of knowledge acquired during the intervention. This t2 questionnaire was again asking to note elements from the intervention in free text fields and querying if the participant was able to apply some of the training content in practice. See Table 3 for the data collected at different points in time.

**Table 3:** Study design and data collection.

Timing	Intervention	Data collection	Items and analysis
On arrival	General instructions and formalities; declaration of consent	Pre-test (t0): questionnaire	Demographics
5 min	Example group: instructional video Lecture group: recorded lecture		
Before simulation	Familiarisation with simulator environment		
5 min	Simulation	Video-recording	Observer coding: team cooperation & medical care
5 min	Debriefing	Audio-recording	(Audio recordings as backup for t1 questionnaires, have not been analysed)
After debriefing		Post-test 1 (t1): questionnaire	Retention of knowledge, evaluation of the training
2 weeks later		Post-test 2 (t2): questionnaire	Retention of knowledge, impact of the training

### Data analysis

To investigate the effects of interventions on group interactions during the simulations, all video recordings were analysed using behavioural observation coding. The videos were coded using MAXQDA [36] following the behaviour coding scheme (see Table 4). The coders were blinded to the video that the participants watched before the simulation. Coders saw recordings of the simulations, including a display of the patient’s current vital parameters. Team processes (e.g., calling a team briefing), measures of medical care (e.g., checking pupil reflexes), and events during the scenario itself (e.g., changes in vital parameters or phone calls) were marked and coded by noting a time interval or time stamp in the videos with a resolution of seconds. If behaviours occurred simultaneously (e.g., one team member applying oxygen mask or blood pressure cuff while others exchanging information in the team) each of the behaviours was marked separately in an overlapping way. See Table 4 for the full coding scheme.

**Table 4:** Coding scheme for video analysis.

Category	Code	Description or example	Type
Initiation	Clarifying roles	What is my role, what is yours?	Interval
Initiation	Handover from ambulance	Ambulance role briefs other participants about patients’ status	Interval
Team	Exchange of information	Team is exchanging information (e.g. observations or data from files)	Interval
Team	Calling a team briefing	A team member initiates a team briefing, can be using a keyword or freestyle	Interval
Team	Planning and prioritising	Team discussion: What needs to be done? What is important now?	Interval
Team	Delegating	Tasks are distributed amongst team members. Either directed by a leader or during team briefing.	Interval
Team	Check / asking for consent	After planning or delegating somebody asks for consent	Interval
Team	Call for help	Requesting outside help (e.g. using phone to call senior physician)	Interval
Med. Care	RR	Applying blood pressure cuff or measuring again	Interval
Med. Care	SpO2	Applying SpO2 finger clip	Interval
Med. Care	Pupils	Checking pupil reflexes	Interval
Med. Care	O2	Applying oxygen mask	Interval
Med. Care	NaCl	Attaching NaCl infusion	Interval
Med. Care	Physical examination	Performing physical examinations on the patient	Interval
Med. Care	Conversation with patient	Talking to the patient, anamnesis. This code is given subordinately: if speaking to the patient is part of a specific measure (e.g., explaining that an O2 mask will be put over the patient’s face), this was not coded additionally as conversation.	Interval
Simulation	Ambulance enters	When ambulance role enters video	Time stamp
Simulation	Surgical enters	When surgical role enters video	Time stamp
Simulation	Internal enters	When internal role enters video	Time stamp
Simulation	Phone ringing	Phone ringing until somebody picks up	Interval
Simulation	Phone call	From picking up until putting down	Interval
Simulation	Change of vital parameters 1	Heart rate 80 -> 100	Interval
Simulation	Change of vital parameters 2	Heart rate 100 -> 50	Interval
Simulation	2^nd^ phone call = end of simulation	Phone starts ringing	Time stamp

Categories were predefined by available options in the simulation environment (Med. Care), by the simulation sequence (Simulation), or by the content of the instructional videos (Team). The behavioural coding scheme was first drafted and then refined during nine pretesting runs. The initiation of the team during clarifying roles when entering the simulation and the initial handover from the participant in the ambulance role have been coded as separate category (Initiation). This first phase of orientating and warming-up for the participants has not been included in the analysis.

Inter-rater reliability was validated for 10% of the sample (n = 5 videos) with a result of a Cohen’s κ = .82 for attributed behaviours and an intra-class correlation (two-way mixed, absolute agreement, single measures) of .99 [CI95: .99–1] for starting times and .82 [CI95: .75-.87] for durations, indicating high levels of agreement. Codes were clustered into the categories “team behaviour”, “medical care” and “simulation” for events that were descriptive for the simulated scenario. The coding scheme is provided in table 4.

The post-test questionnaires contained free-text fields to assess which of the training contents the participants remembered. Answers were mostly formulated as keywords; one researcher counted them when matching to the team behavioural codes. Video coding data and the questionnaires (see Table 3) were consolidated using an Oracle database and analysed using R [37].

To test our main research question, the study groups were compared regarding the occurrence of team behaviour using χ^2^ tests, i.e., if a group engaged in planning and prioritising. Time spent engaging in team behaviours was compared using t-tests for non-paired group differences. Group differences concerning training evaluations were tested using Mann-Whitney-U tests. Recall of training content was tested using a mixed-design analysis of variance. To control for interference between team processes and medical care, group differences were compared respectively. The alpha level was set to *p* = .05 for all statistical tests. To correct for multiple testing, Bonferroni-Holm correction was conducted within the tested subsets.

## Results

### Participants

A total of 129 participants were recruited for this study. Six participants were excluded from the analysis because they were students who filled in for participants who did not show up for their session. These students were from the same cohort as the other participants but were affiliated with the simulation centre. They were instructed about standard behaviour in the ambulance role, because this team member could go to a passive role in the simulated scenario as a realistic behaviour, and evenly distributed between example group (n = 3) and lecture group (n = 3). Another complete group of three participants had to be excluded because one team member revealed during the simulation that she was not a medical student, potentially biasing group behaviour. This resulted in a final study sample of 120 participants. The participants’ demographics are shown in Table 5.

**Table 5:** Demographics of study participants.

	Example group	Lecture group	Statistical test for group differences
Number of participants / number of groups	63/22	57/20	*p* > .999, χ^2^ [120] < 20.66
Sex (male/female)	20/43	17/40	*p* = .989, χ^2^ [1] < 0.01
Age (mean/range)	25.2/19-33	23.8/18-34	*p* = .009, *t*(119) = 2.65
Study semester (mean)	7.9	7.9	*p* = .911, *t*(119) = 0.11
Percentage of participants who had already completed their preliminary medical examination	85%	83%	*p* = .716, χ^2^ [1] = 0.13
Percentage of participants with relevant previous professional experience	25%	17%	*p* = .335, χ^2^ [1] = 0.93

### Group comparison

The groups were compared on the following measures: behaviour during the simulation (team cooperation and medical care), retention of knowledge from training contents, and results of the evaluation. Means, standard deviations, and the results of the group comparisons are shown in Table 6.

**Table 6:** Group comparison regarding time allocation during the simulation, perception, and learning.

	Example group *M*(*SD*) *n*	Lecture group *M*(*SD*) *n*	*Test statistic*	*p-*value^a^	Cohen’s *d*
**Timings of team processes during simulation**					
Duration of team coordination overall (in sec)	135(45) *n* = 22	77(33) *n* = 20	T = 4.82 df = 38.6	.002*	1.31
Start of first team briefing (in sec)	76(32) *n* = 22	99(79) *n = 20*	T = 1.20 df = 24.6	.964	0.37
Duration of information exchange (in sec)	80(34) *n* = 22	45(30) *n* = 20	T = 3.57 df = 40.0	< .001*	1.10
Duration of planning and prioritising (in sec)	26(17) *n* = 22	14(10) *n* = 19	T = 2.65 df = 34.9	.036*	0.83
Duration of delegating (in sec)	12(14) *n* = 19	9(8) *n* = 13	T = 0.90 df = 27.8	>.999	0.33
**Timings of medical care during simulation**					
Duration of medical care overall (in sec)	74(25) *n* = 22	101(31) *n* = 20	T = 3.10 df = 36.8	.003*	0.95
Duration spent talking to the patient (in sec)	58(29) *u* *n* = 22	72(39) *n* = 20	T = 1.36 df = 34.6	.368	0.42
First supply of supplemental oxygen (in sec)	243(62) *n* = 18	247(45) *n* = 18	T = 0.21 df = 31.1	>.999	0.07
First call for help (in sec)	211(40) *n* = 14	215(33) *n* = 7	T = 0.22 df = 14.5	>.999	0.10
**Participants’ evaluation of the training** Rated on a 6-point Likert scale (scores ranged from 1–6, with 6 indicating the strongest approval of the statement)					
Scenario was relevant for practice	5.37(0.85) *n* = 61	5.31(0.72) *n* = 57	U = 1964	>.999	-
Scenario was realistic	4.84(1.02) *n* = 62	4.88(0.94) *n* = 57	U = 1835	>.999	-
Training content was useful for daily routine	4.63(1.05) *n* = 62	4.50(0.93) *n* = 57	U = 2077	.969	-
Video was helpful during the scenario	4.53(1.33) *n* = 61	3.55(1.32) *n* = 57	U = 2610	< .001*	-
Team approach during the scenario was structured	3.53(1.32) *n* = 56	3.08(0.87) *n* = 49	U = 1748	.120	-
I had a clear concept what to do during the scenario	3.33(1.21) *n* = 56	3.20(1.08) *n* = 49	U = 1556	>.999	-
**Learning: retention of knowledge from training content**					
Elements of CRM tool remembered at t1 (after debriefing)	2.40(0.89) *n* = 62	1.15(1.02) *n* = 57	T = 7.20 df = 116.9	< .001*	1.30
Elements of CRM tool remembered at t2 (> 2 weeks later)	1.87(1.24) *n* = 39	0.77(0.92) *n* = 43	T = 4.54 df = 69.7	< .001*	1.00

^a^Bonferroni-Holm corrected

* *p* ≤ 0.05 (statistically significant)

The groups did not differ regarding previous professional experience (see Table 5). We also surveyed participants regarding previous team trainings with focus on team procedures they had participated in. There were also no group differences (chi^2^(1) = 0.08, p = .774): For the example group, 49% reported partaking in such a training prior to our intervention and for the lecture group, 55% reported experiencing a similar training. Previous team training experience and previous professional experience were not associated with any of the independent variables we measured.

#### 1. Behaviour during the simulation

Both groups engaged in team processes (i.e., exchanging information, planning and prioritising tasks) at least once during the simulations. On average, the teams applied 3.14 out of 4 (*SD* = 0.75) aspects of team cooperation that were taught in both video instruction conditions. However, as shown in Table 7, more teams in the example group tended to show team behaviours characterising the content of the CRM tool. The difference was statistically significant for the explicit call-out of the team briefing (in the example group “Team Check”, in the lecture group the “Stop” word). The example Group A showed an average of 3.5 of the 4 aspects of team coordination that were taught to both groups, while the lecture group showed 2.75 aspects; this difference was statistically significant (*T* [39.9] = 3.71, *p* = .001, *d =* 1.5).

**Table 7:** Absolute occurrence of team cooperation behaviour and medical care during the simulations.

	Example group	Lecture group	χ^2^ (df)	*p*-value^a^
**Team cooperation**				
Explicitly calling-out a team briefing	68%	15%	10.05 (1)	.001*
Exchanging information	100%	100%	-	-
Planning and prioritising tasks	100%	95%	<0.01 (1)	>.999
Delegating tasks	82%	65%	0.79 (1)	>.999
Asking for consent^1^	32%	5%	3.30 (1)	.138
^1^ This aspect was demonstrated only in the video for the example group				
**Medical care**				
Supplemental oxygen	82%	90%	0.10 (1)	>.999
Blood pressure	100%	100%	-	-
Pulse oximetry	100%	100%	-	-
NaCl infusion	36%	40%	0.00 (1)	>.999
Pupil reaction check	73%	85%	0.35 (1)	>.999
Physical examination	68%	85%	0.84 (1)	.720
Call for help	64%	35%	2.39 (1)	.122

^a^Bonferroni-Holm corrected

The groups did not significantly differ in terms of the number of medical measures that were conducted, time spent talking to the patient, time of the first supply of supplemental oxygen, or time of the first call for help (see Table 6). Comparing the application of medical measures separately also revealed no difference between groups (see Table 7).

**Fig 3:**
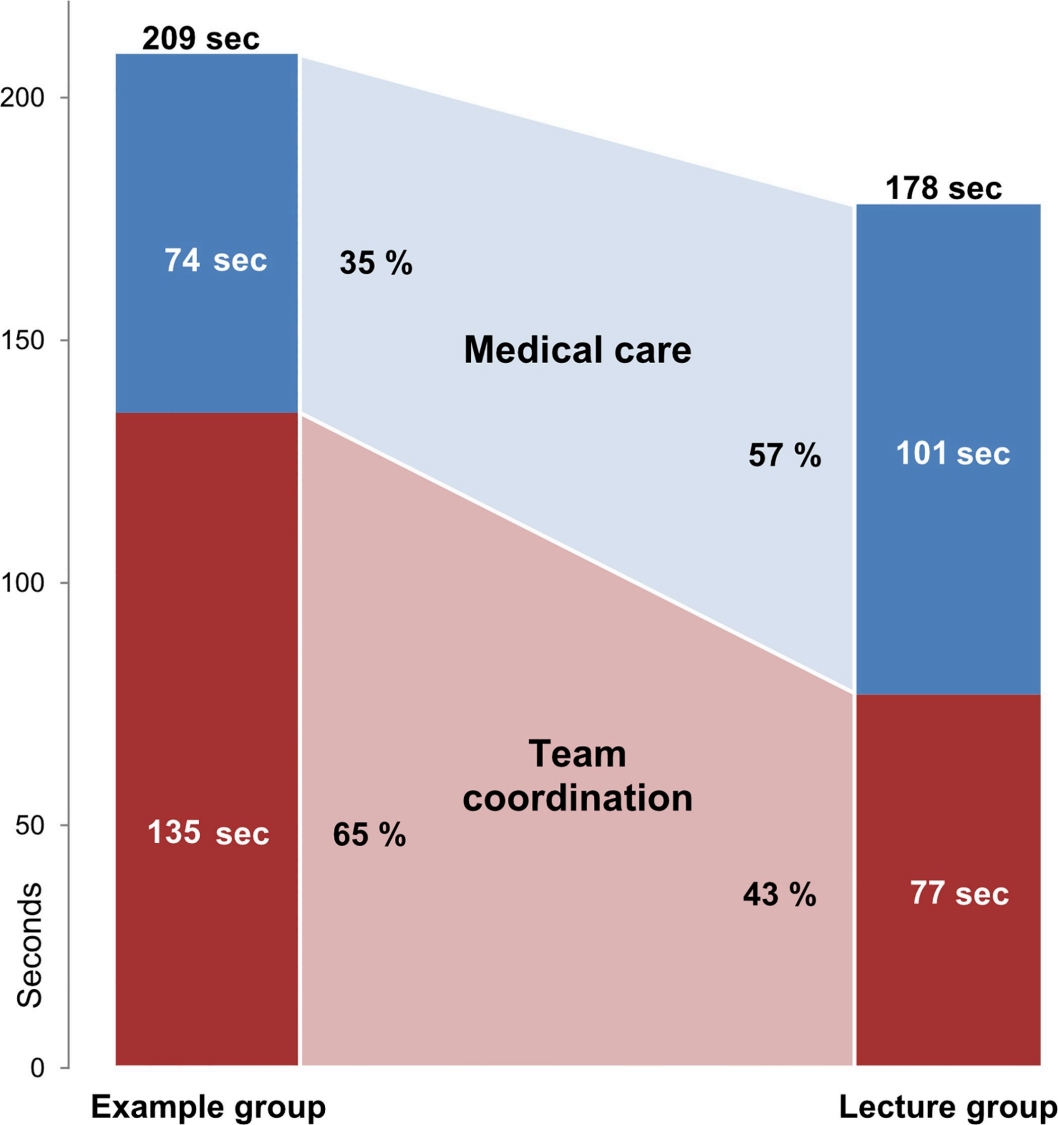
Comparison of cumulative time coded for medical care and team coordination. The example group spent almost twice as much time coordinating the team compared with the lecture group. The example group also cumulated more time intervals of actions and team behaviours.

Comparison of how the groups allocated their time in the simulator revealed that the example group spent significantly more time coordinating the team overall. With 135 seconds they devoted almost twice as much time exchanging information, planning and prioritising compared with the 77 seconds the lecture group spent for team coordination. The cumulative amounts of time coded during the simulations are shown in Fig 3, grouped for medical care and team coordination. For the example group, the sum of the coded time intervals was overall higher than for the lecture group (t[32] = 2.30, p = .03, d = 0.70). This can be observed as all aspects of the groups’ behaviour were coded separately and overlapping if activities occurred at the same time (see data analysis section).

#### 2. Retention of knowledge from training content

Table 6 demonstrates how participants remembered the training content at t1 and t2. Directly after training, the example group remembered significantly more elements of the CRM tool that were presented in the videos than the lecture group. The effect was even larger 2 weeks after the training. Both groups tended to recall less after this time (F[1] = 10.91, *p* = .001, η_partial_ = .12 for the main effect; F[1] = 1.04, *p* = .312, η_partial_ = .01 for the interaction).

#### 3. Evaluation of the training

Both groups rated the scenario as relevant for practice (85% of participants rated as 5 or 6 points on the 6-point Likert scale) and realistic (67%), and the training content was considered useful for daily routines (53%). Table 6 shows a comparison of participants’ ratings of the videos. Members of the example group perceived the video as more helpful.

During the survey 2 weeks after training, participants were asked if they were able to apply techniques they were taught during the training. In the example group, 10 of 47 (21%) members answered this question with “yes”, compared with five of 51 (10%) members of the lecture group. This difference was not statistically significant (χ^2^ [1] = 2.48, *p* = .115).

## Discussion

Both groups exhibited most of the behaviours included in the instructional videos during the simulations and were able to recollect them 2 weeks later. This could be descriptive evidence of the feasibility of microlearning in this context. Participants generally considered the intervention realistic and helpful.

Observer coding of behaviour in the simulator provided a detailed analysis method, enabling us to closely examine how groups allocated their collective time during a critical situation. In addition, the between-groups design effectively demonstrated the differences caused by changing the training method. The current findings highlight the importance of “how” to train in the context of CRM, identifying potentially useful areas for optimisation of educational programs.

The example group, who watched the Team Check video, demonstrated significantly more of the trained content during the simulation. This group had better retention two weeks after the intervention. They also reported that the tool was more helpful. Although the example group spent more time on team coordination, the teams did not significantly differ in the number of executed medical measures. Thus, in the current study, the didactic concept of the instruction caused a difference between the learning success of the groups.

Also, the example group tended to initiate the first team briefing earlier (defined as the first exchange of information after the handover) and to spend more time delegating tasks. Both of these mean differences showed small to medium effect sizes but did not reach a level of statistical significance. The lecture group spent significantly more time on medical care. They also tended to spend more time talking to the patient. Even if these differences did not reach statistical significance, they showed medium to large effect sizes (see Table 6) and should be validated in future studies with larger samples.

The results indicate that a discrete CRM tool similar to a structured team briefing can be learned in a 15-minute intervention. Thus, it appears feasible to design a training intervention encompassing all components of ELT in this short time span. Participants demonstrated newly-acquired competencies in the simulation and recalled them several weeks later. In addition, participants reported the format to be helpful. These findings are comparable with those of Weller et al., who evaluated the SNAPPI call-out method using simulations 4–6 weeks after participants saw a short video instruction [8].

### To what extent does the instructional approach during a module affect immediate learning outcomes and retention of knowledge?

The example group had better retention of training contents immediately after and 2 weeks after the intervention. This group also considered the tool to be more helpful. Although both groups had the same exposure time, the results revealed that the instructional video showing an example of good CRM practice was significantly more effective regarding knowledge retention, but also with regards to its immediate impact on participants’ behaviour during the simulation scenario than a traditional lecture on the same topic. The potential of applying psychological and educational expertise to the design of academic lessons, even for very theoretical subjects, was demonstrated in a previous study of students in a physics class, who performed significantly better when taught in small groups and interactive courses, compared with a traditional lecture [26].

Medical care in its application and especially communication and decision making in critical situations are practical tasks that require procedural knowledge as opposed to academic considerations and conceptual (theoretical) knowledge [38]. The interventions for our two groups contained the same topics but differed in how a lecture versus role players framed content into a concrete medical setting. In a similar vein, Semler et al. demonstrated that showing a 12-minute video of a simulated emergency, exemplary managed by faculty staff, was similarly effective to participating in a simulation and more effective than being presented teamwork principles by traditional didactic slide presentation [39]. Our example group received both a concrete example in a medical context and a simulation experience. The results can be interpreted that specificity of content and training opportunity might be a key factor to acquiring procedural knowledge for teamwork situations.

### Does an increased emphasis on team processes concur with measures of medical care?

We found a clear difference in how the groups allocated their time during the simulation. The example group spent more time on team coordination processes, such as exchanging information and planning actions, and less time on actual measures of medical care with the patient. Among all possible measures in the simulator, the application of supplemental oxygen and call for assistance by a senior physician (“call for help”) were the essential and urgent actions in the given situation. We observed no difference between the groups in those crucial actions.

Importantly, an increased focus on team processes must not compete with practical measures of care. When cumulating the times spent on team coordination and patient treatment, the example group spent more time engaged in both of these activities. As the example group coordinated their team while simultaneously preparing the patient treatment, they were able to undertake more activities in the same time, compared with the lecture group. Similar observations have previously been reported by studies of the clinical performance of teams during critical events [40,41] and research on CRM training from a learning perspective [42].

### Limitations

One weakness of the study is related to the recruitment of medical students as a study group. Because the cohort of students dispersed after the intervention, a longitudinal study of the personal and organisational impact of training was not possible. However, the current study sample was chosen deliberately to avoid spill-over effects from previous team trainings or leadership experience.

Furthermore, the study was designed as an A/B comparison without a direct control group. This design avoided ethical concerns that may have arisen from exposing participants to a critical situation without any preparation, or with irrelevant instructions. Also, the two videos differed in more than one way. Team Check and 10-for-10 are similar in content but put in different tools. Additionally, one was presented as example video, the other as lecture. As the training participants received during this study was focused on only one aspect of teamwork, we are limited in our ability to generalize whether the simulation behavior differences would extend to more diverse clinical scenarios as well.

Third, this study design is unable to answer the question to what extent CRM-like behaviour might be natural behaviour in a social group that participants might had exhibited during the simulation even without a prior intervention. The team behaviour “Asking for consent” could be considered as control variable in this regard. This aspect was not part of the lecture group’s video but was included in the Team Check protocol. Only 5% of the lecture group teams demonstrated this behaviour during the simulation by themselves, while 32% of the Team Check teams exhibited this behaviour after they saw it in the instructional video (see Table 7). This group difference reached a medium effect size but did not reach statistical significance due to the small sample size. Even though groups did not differ regarding experiences with team trainings or relevant professional experience, we cannot say with certainty to what extent the tested behaviour would have occurred regardless of the training.

### Meaning of the study: Possible implications for clinicians and policymakers

We adopted the concept of microlearning to describe a relatively short and standardised intervention. The current results support an idea worth further investigation: by modularising CRM training content into smaller chunks and using professional instructional videos, a curriculum can be decomposed into increments of a manageable size. Lightweight incremental training modules could ameliorate the effects of understaffing in hospitals when used to blend learning into daily staff routines, as microlearning portions.

Finally, the current study contributes to the “science of training” in CRM and patient safety. The differences we observed between the effectiveness of an academic lecture compared with an action-oriented instructional video indicate further potential for enhancing the impact of training.

### Unanswered questions and future research

The current study did not utilise the full potential of microlearning. Smartphone apps, as well as interactive and self-directed content in modern e-learning should be embraced to foster the educational landscape in patient safety. Further experiments and developments in this field are necessary.

It should be noted that the ELT cycle operates as a spiral, in the sense of a recurring process. The current study presented a single run through the ELT cycle at best. However, a cascading training design involving multiple modules can be expected to have further benefits. It may be useful for future research to evaluate a curriculum of multiple incremental training interventions.

Given that all participants in the current study were medical students, it is important to evaluate the degree to which our findings can be translated to the education of experienced healthcare staff. In addition, the organisational context of the hospital in which such CRM interventions are to be embedded should be investigated to examine the potential influence on training success.

## Supporting information

S1 File Video interventions. Transcript of the instructional video (example group) and videotaped lecture (lecture group).
(DOCX)

S2 File Survey questions. Questions and answering options administered at t0, t1,and t2.
(PDF)
