# Supplementary material for: Microlearning for patient safety: Crew resource management training in 15-minutes
Source: PLoS One. 2019 Mar 7;14(3):e0213178. doi: 10.1371/journal.pone.0213178 (PMC6405193; doi:10.1371/journal.pone.0213178)
Supplement: S1 File — Transcript of the instructional video (example group) and videotaped lecture (lecture group). (DOCX) [file pone.0213178.s001.docx]

**Supporting Information: Video Interventions**

Transcript of the instructional video (example group) and videotaped lecture (lecture group).

**Example group (instructional video)**

| 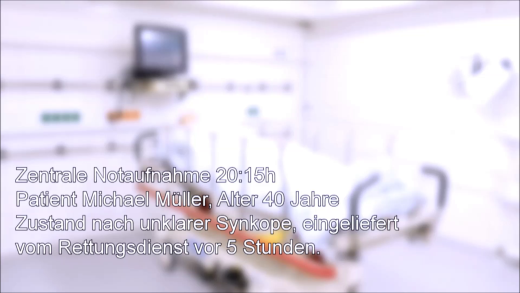 | [0:00] Video starts with a blurred image of a hospital room. Text overlay:  “Emergency department 8:15 pm  Patient name, age 40 years  Condition after syncope  Admitted by ambulance service 5 hours ago” |
| --- | --- |
| 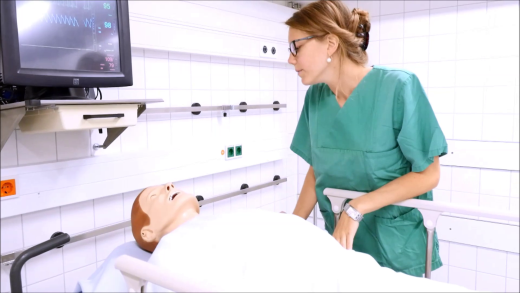 | [0:08] Medical student enters the room. Patient is lying in a bed, ECG and pulse oximetry are displayed on a monitor.  Short pause and voice from off-camera: “There are situations that can become critical very quickly. Whenever that happens, it is important to focus all information and resources within the team.”  Student speaks to the patient in order to take a blood sample. Patient is somnolent and does not give appropriate answers.  Student activates the alarm button on the patient’s monitor and tends to the patient. |
| 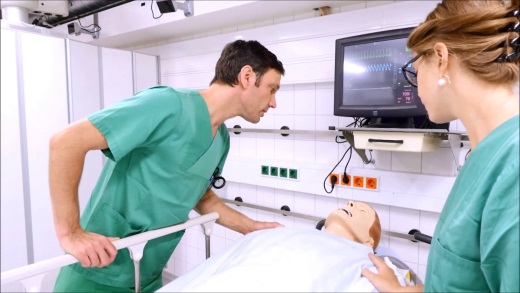 | [0:42] Senior nurse enters the room and asks about the reason for the alarm.  Student reports her observation: the patient was somnolent and exhibited an increased heart rate.  Nurse briefly interacts with patient, then asks student to page the physician on duty. |
| 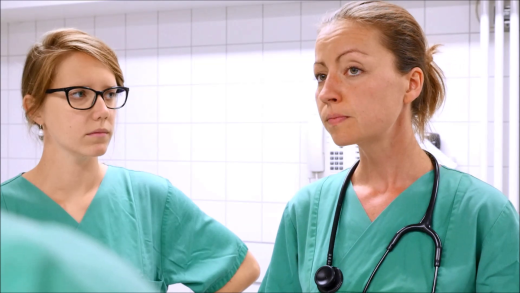 | [1:05] Physician enters the scene. Nurse briefly describes the situation. Physician answers: “Ok, let’s do a Team Check so that everybody knows what to do.”  The team steps back to the foot of the patients’ bed. |
| 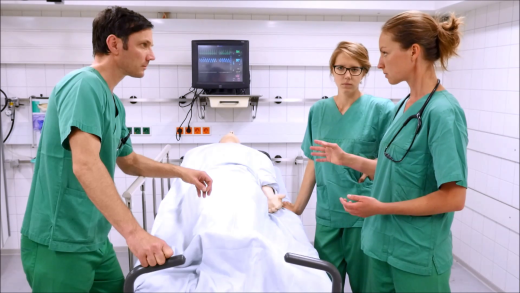 | [1:20] Physician initiates the Team Check protocol: “What is the situation?” Nurse reports that the patient was admitted by the ambulance service several hours ago. Student reports that she entered the room to take the blood sample and found the patient somnolent. Physician states that her shift started only recently and confirms that this is a patient whose radiological results were not yet available. She then summarises the situation to provide a common understanding and asks everybody if the summary was correct.  [2:14] Physician prioritises the necessary actions, and asks the team if she’s forgotten anything.  [2:25] Student suggests double-checking the patient for physical injuries. Physician approves of the idea.  [2:32] Physician delegates actions to all team members then asks team to consent again, ends Team Check procedure with “go”. |
| 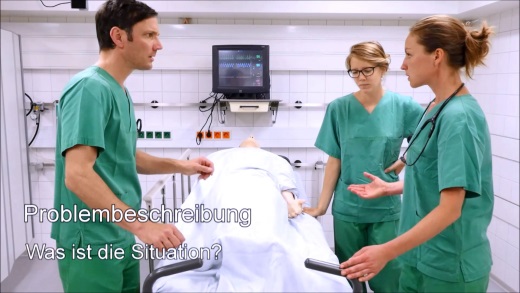 | [3:05] Pause, and voice from off-camera: “This situation was managed well. Let’s recap in detail what happened here …”  Voice then lists the three steps of a Team Check; the corresponding scene from the previous video is played again after each step.  For each step a text overlay displays the name of the specific step and the leading question. |
| 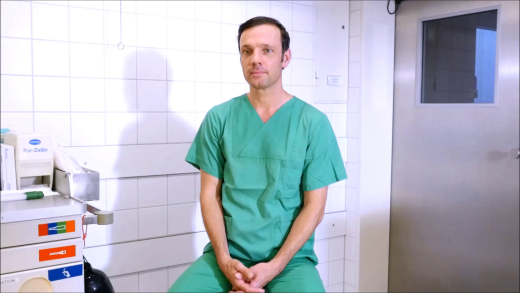 | [4:06] Interview setting: the three characters explain why they consider the Team Check useful. Physician: to consolidate and focus sources of information and to thus avoid mistakes.  [4:20] Nurse: to reduce stress in teamwork situations.  [4:30] Student: because a student can be integrated in the team and is encouraged to speak up. |
| 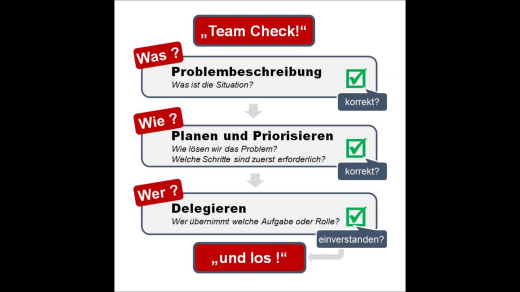 | [4:40] Team Check procedure displayed as a diagram in a still image. |
|  | [5:00] End |

**Lecture group (recorded lecture)**

| 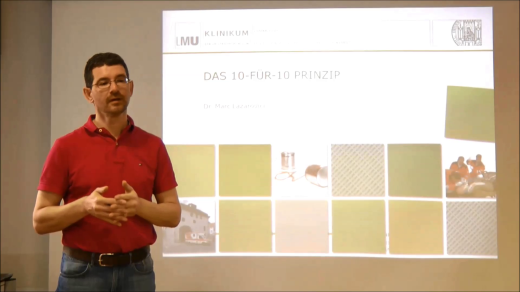 | [0:00] Video starts with Lecturer welcoming audience. The purpose of the lesson is to demonstrate a concept for team interactions called “10-for-10” or “team-time-out”. |
| --- | --- |
| 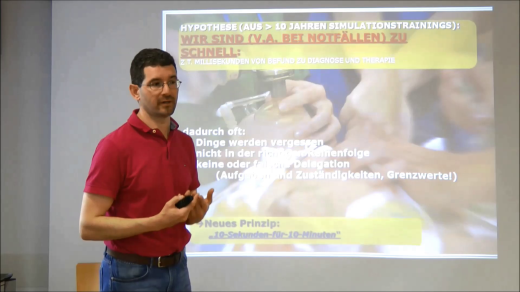 | [0:13] Presents insights from more than 10 years of experience in healthcare simulation training, supporting the need for a concept of team interaction:  Time from perception to action passes far too quickly in critical situations.  This has negative results:   - Things are overlooked - Wrong line of actions - No or wrong delegation   The reason for this is not incompetence on the individual level, but a lack of coordination at the team level under time pressure. |
| 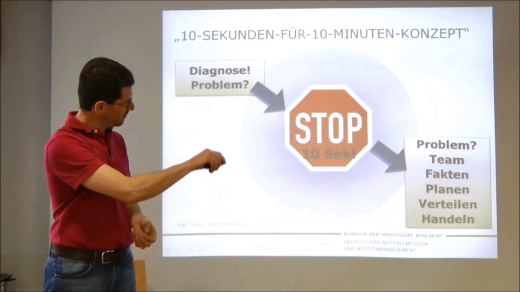 | [1:55] Introduces the “10-for-10 principle”:   - Whenever a problem occurs or a diagnosis has been made, the team leader calls out “stop” to get the team’s attention. Only life-saving measures like CPR* are to be continued. - Team leader summarises current problem and known facts - Names everybody who is on the team - Plans next steps - Delegates tasks - Then team is to go on with actions.   * cardiopulmonary resuscitation |
| 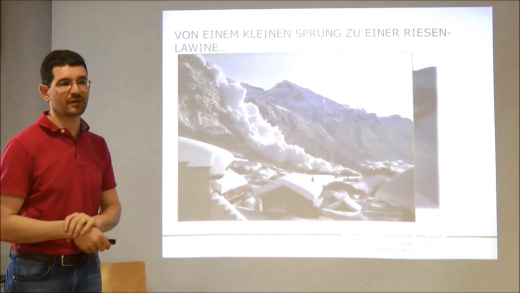 | [3:13] Explains the need to go through a 10-for-10 briefing more than once, using concept of avalanche protection with multi-layer fences as an example. Small barriers can prevent a large avalanche from unfolding that cannot be stopped once underway. |
| 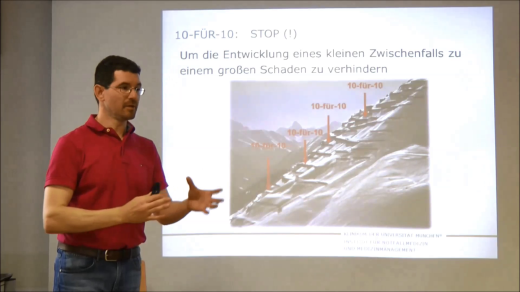 | [3:50] Frequent team briefings can help to avoid minor misunderstandings or errors before they build up and cause serious harm. |
| 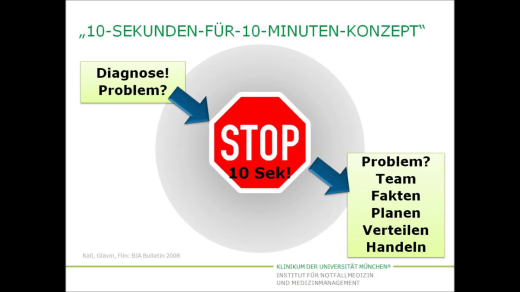 | [4:15] 10-for-10 procedure is displayed as a diagram. |
|  | [5:00] End |
