# Supplementary material for: Microlearning for patient safety: Crew resource management training in 15-minutes
Source: PLoS One. 2019 Mar 7;14(3):e0213178. doi: 10.1371/journal.pone.0213178 (PMC6405193; doi:10.1371/journal.pone.0213178)
Supplement: S2 File — Questions and answering options administered at t0, t1,and t2. (PDF) [file pone.0213178.s002.pdf]

Supporting Information: Survey questions

Question or item	Value
**Pre-test questionnaire at t0:<br>Demographics**	
Semester of study	number
First state examination completed?	yes / no
Clinical traineeships completed?	yes / no
Previous relevant job experiences other than medical studies?<br>(e.g. vocational school, emergency medical service)	yes / no
If previous question was answered with "yes"<br>Please describe your previous job experience	free text field
Have you participated a team training before?	yes / no;<br>if yes: free text field
If previous question was answered with "yes"<br>Please describe your previous team training experience	free text field
Sex	male / female
Age	number
**Post-test questionnaire at t1, immediate after intervention:<br>Retention of knowledge and evaluation of the training**	
The scenario was relevant for practice	6-point Likert scale
The scenario was realistic	6-point Likert scale
The training content was useful for daily routine	6-point Likert scale
The video was helpful during the scenario	6-point Likert scale
Our team approach during the scenario was structured	6-point Likert scale
I had a clear concept what to do during the scenario	6-point Likert scale
You watched a video at the beginning. What elements of this video can you recollect?	free text field
Did you hear before about the concept for team briefings that we presented today?	Yes, university / yes (free text field) / no
Have you previously encountered a team briefing in a critical situation in practice?	yes, often / yes, seldom / no
If previous question was answered with "yes"<br>Please describe the context such situations	free text field
**Post-test questionnaire at t2, two weeks after intervention:<br>Retention of knowledge and impact of the training**	
What can you remember oft the training you participated with us?	free text field
You watched a video at the beginning. What elements of this video can you recollect?	free text field
In the video a concept like „Team-Check“ or „10 for 10“ was demonstrated. Please describe this concept as far as you remember.	free text field
Where you able to apply anything you learned during the training in practice?	yes / no
If previous question was answered with "yes"<br>What?	free text field
Do you feel like the training was beneficial to you?	yes /some / little/ no
If previous question was answered with "yes" or "some"<br>What?	free text field
